# Serum Chemokine CXCL7 as a Potential Novel Biomarker for Obstructive Colorectal Cancer

**DOI:** 10.3389/fonc.2020.599363

**Published:** 2021-02-10

**Authors:** Longhai Li, Lihua Zhang, Ting Zhang, Xiaowei Qi, Gang Cheng, Lingxia Xia

**Affiliations:** ^1^ Department of Science and Education, The People’s Hospital of Bozhou, Bozhou, China; ^2^ Department of Pathology, The Affiliated Hospital of Jiangnan University, Wuxi, China; ^3^ Department of Pathology Cancer Research Center, The Affiliated Hospital of Jiangnan University, Wuxi, China; ^4^ Department of Oncology, The People’s Hospital of Bozhou, Bozhou, China

**Keywords:** CXCL7, biomarker, obstructive colorectal cancer, diagnosis, prognosis

## Abstract

Due to the lack of typical symptoms and signs and sensitive indicators for early diagnosis of obstructive colorectal cancer (OCRC), it is critically needed to find new novel biomarkers to ameliorate the management of OCRC patients. In this study, 472 blood samples were collected and measured by enzyme-linked immunosorbent assay (ELISA) to investigate the value of serum chemokine ligand 7 (CXCL7) in diagnosis and prognosis for OCRC patients. The median concentrations of CXCL7 in non-OCRC and OCRC were both higher than that in controls (both *P* < 0.05). Importantly, the median serum concentration of CXCL7 in OCRC was also higher than that in non-OCRC (*P* < 0.001). In all OCRC patients, the area under the curve (AUC) of CXCL7 was 0.918 with a sensitivity of 86.54% and a specificity of 81.87%. Similarly, the AUC of CXCL7 was 0.684 when the diagnostic test was performed between OCRC and CRC patients. CXCL7 had a higher AUC than other markers. The concentration of CXCL7 in 40 postoperative OCRC patients was higher than normal people and lower than preoperative patients. The median survival time was 62.00 months and the 5-year overall survival (OS) rate of the patients was 51.80% in all 155 OCRC patients. Multivariate Cox proportional hazard regression model analysis showed that high CXCL7 in serum was independent factors associated with poor OS of OCRC patients (HR = 2.216, *P* = 0.032). These results demonstrate that serum CXCL7 may be a potential biomarker both in diagnosis and prognosis for OCRC patients.

## Introduction

Colorectal cancer (CRC) is one of the most common cancer in the digestive system worldwide, and the morbidity and mortality are at the forefront. Nowadays, CRC remains the most fatal cancer in China (1–4). According to the newly published cancer data report of the United States in 2017, the number of CRC patients and deaths were 135,430 and 50,260 in China, which ranked the top of the gastrointestinal cancer both in morbidity and mortality (3). Similarly, in a survey report of China in 2015, the number of CRC patients and deaths were 376,300 and 191,000 respectively, followed by gastric cancer, liver cancer, and esophageal cancer (5). CRC patients can have a higher survival rates if having an early diagnosis and fit treatment (6, 7). About 8 to 29% of CRC patients were obstructive colorectal cancer (OCRC) when diagnosed at the first time (8). Acute colonic obstruction often needs emergent surgical management to decompress the patient’s intestine, so the surgical resection becomes the most important curative treatment for OCRC (9). OCRC is also very difficult to definitely diagnose in the early stage due to lack of its early typical symptoms and signs and sensitive indicators for early diagnosis (10). Although the diagnosis and treatment have been improved in recent years, this emergency surgery still exists with high morbidity (40–50%), mortality (15 to 20%), and stoma creation rates compared with the non-OCRC patients (11), which could affect the life quality of OCRC patients. Therefore, OCRC patients often have advanced stages and worse long-time survival compared with non-OCRC patients (8–11), for the reason of high invasiveness and distant metastasis (12).

Chemokines, 8–12 kDa secretory proteins, can regulate the migration of leukocyte and play vital roles in many physiological and pathological processes, including inflammation and repairing damaged and wound tissues (13–15). These small proteins can be classified into CXC-, CX3C-, CC-, and XCL subgroup chemokines based on the position of conserved cysteines near the N-terminus (16). CXCL7, also named NAP-2, which belongs to CXCL-subgroups, is released by activated platelets and is related to the occurrence and development of various tumors (17–21).

Nowadays, OCRC can be diagnosed by some invasive and non-invasive methods. However, these methods may not be fully implemented sometimes because of following pain and low sensitivity (22, 23). Therefore, it is critical to detect OCRC before it advanced and reduce the chance of recurrence and physical and mental harm. OCRC was diagnosed for emergency intestinal obstruction and postoperative pathological diagnosis, so it lacked signs for early diagnosis (24). Our previous study has pointed out that serum CXCL7 may be an auxiliary diagnostic biomarker for CRC (17). However, the role of serum CXCL7 in OCRC diagnosis had not been illustrated, and the concentration had also not been detected.

In this study, 472 people were recruited, including 156 OCRC patients, 156 non-CRC patients, and 160 healthy people. Then, all of the blood samples were collected and measured by enzyme-linked immunosorbent assay (ELISA). Meanwhile, each person^’^s data of biomarkers (CEA, CA125, and CA19-9) were also acquired in the Clinical Laboratory. Moreover, overall survival time and state of 155 OCRC patients were obtained after surgical operation. ROC curve analysis and logistic regression models were adopted for exploring the diagnostic ability of serum CXCL7 in OCRC patients. Kaplan–Meier plotter and Cox proportional hazard regression models were used to explore whether serum CXCL7 could be a prognostic marker or not. Importantly, we try to explore the use of CXCL7 in OCRC patients’ diagnosis and investigate the long-term oncologic outcomes and prognostic factors in OCRC patients. With the analysis, we found that the median serum CXCL7 in OCRC was also higher than that in the non-OCRC group and also higher than in controls, which was consistent with our assumption that the serum concentration of CXCL7 in OCRC patients was highly expressed. Systematic analysis showed that CXCL7 in serum may be a preferable diagnostic biomarker for OCRC patients. Furthermore, serum CXCL7 is identified as an independent factor with poor prognosis in OCRC patients [Serum CXCL7 is not an independent factor in non-OCRC patients (data not shown)]. All in all, serum CXCL7 may be a potential biomarker both in diagnosis and prognosis for OCRC patients.

## Materials and Methods

### Subjects and Serum Sample Selection

Blood samples from 156 OCRC patients, 156 non-OCRC patients, and 160 healthy people were collected from the department of Cancer Research Center in the Affiliated Hospital of Jiangnan University from May 2012 to May 2018. The selection criteria of non-CRC patients and healthy people were selected according to our previous study. The OCRC patients were chosen according to the following criteria. (1) Patients had clinical symptom, physical examination, imaging examination (such as abdominal computed tomography, CT), laboratory examination, colonoscopy, and surgical findings. (2) Postoperative pathological diagnosis conformed to the clinical judgments. (3) Other conditions were referred to the rules of our previous study (16). Informed consents were obtained from all 472 subjects in this study. The study was approved by the Institutional Research Ethics Committee of the Affiliated Hospital Jiangnan University.

The blood samples of all 312 CRC people were collected in the morning within 3 days before surgery. The blood samples of 160 healthy people were also obtained in the morning. The samples were centrifuged at 2,000×g for 20 min, then subpackaged serum. Part of the serum of each sample was used to detect the levels of CEA, CA125, and CA19-9. The other subpacked serum of samples were used to measure the concentration of serum CXCL7.

### Enzyme-Linked Immunosorbent Assay

The levels of serum CXCL7 was quantitatively measured by using the NAP-2/CXCL7 detection ELISA kit (Shanghai Langdon Biotechnology; Shanghai, China). Manipulation steps were conducted according to the manufacturer’s instruction. Specific steps were the same as our previous study (15). Each index of all samples was repeated twice. Reference lines for CEA, CA125, and CA19-9 were identified as 5.0 ng/ml, 35.0 U/ml, and 37.0 U/ml.

### Postoperative Follow-Up and Data Collection

The clinical and follow-up data of OCRC patients were retrospectively collected. The data were as follows: (1) Demographic data: gender and age. (2) Pathological data: tumor location, colon cancer site, tumor size, histological type, T stage, N stage, M stage, TNM stage, vascular invasion and nerve invasion. (3) Hematology index: CXCL7, CEA, CA125, and CA19-9 in serum. The collection methods of follow-up data: A systematic follow-up was carried out for OCRC patients. A follow-up team was set up, which was composed of three persons, with one person as the team leader, responsible for the formulation of follow-up plan, data collection and task coordination; the other two persons as follow-up personnel, mainly responsible for the collection of the follow-up data of OCRC patients. The follow-up time was defined as once every 2 months in the first year after operation, once every 3 months in 2nd–4th years, and once every 6 months in 5th years and above. The follow-up methods were outpatient reexamination follow-up, letter follow-up and telephone follow-up, using home follow-up and e-mail for special cases. The follow-up contents were as follows: survival status, cause of death, recurrence and cause of tumor, and whether metastasis occurred. The follow-up data were collected and checked by two follow-up personnel. As for the disagreement, the team leader could judge the results. The definition of overall survival time (OS) of OCRC: the start time of follow-up was clearly determined once diagnosed, and the end point of follow-up was death caused by specific cause of OCRC (Such data was defined as complete data). The other results of follow-up time were classified as censoring data (without death event, other death caused by non-tumor factors, loss of visit and so on).

### Statistical Analysis

The concentration of serum CXCL7 and four markers in two groups were compared by using Mann–Whitney U-test, and interpretation of results with median (M) and interquartile range (IQR; Q_1_–Q_3_). Kruskal–Wallis H-test was also adopted when the results in three or more groups were analyzed. Categorical variables were described by frequencies (n) and percentages (%) with a statistical test of chi-square (χ^2^) test. To assess the predictive ability of CXCL7 and CEA, CA125, and CA19-9 for OCRC, the logistic regression models were used to optimize the diagnostic efficiency. ROC curves were used to estimate the diagnostic value, including the area under the ROC curve [AUC, 95% confidence interval (CI)], Youden index (sensitivity +specificity-1) and Interactive dot diagram. The overall survival time (OS) of OCRC patients was estimated by using the Kaplan–Meier analysis, and log-rank test was used for statistical difference test. Univariate and multivariate Cox proportional hazard regression models were used to evaluate the independent prognostic factors. All analyses were performed by using the SPSS 21.0 statistical software (IBM; Armonk, NY, USA), and *P <*0.05 was statistically significant.

## Results

### Median Serum Levels of CXCL7 and Tumor Associated Antigens (CEA, CA125, and CA19-9) in Patients With Non-OCRC, OCRC, and Controls Prior to Surgery

**Figure 1:**
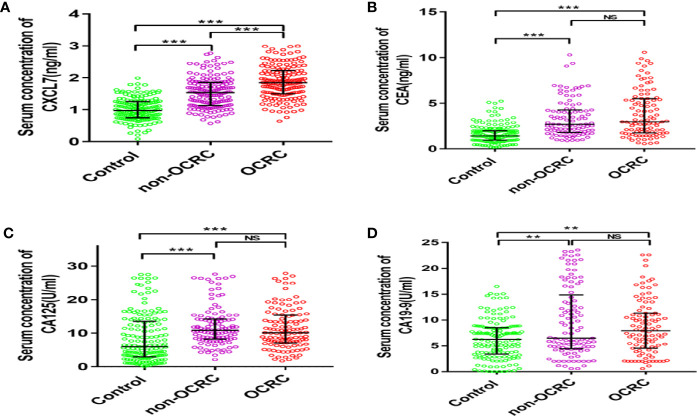
Comparison of the expression of serum CXCL7 and tumor-associated antigens in patients with non-OCRC, OCRC and health control group. **(A)** Serum CXCL7 (Kruskal–Wallis test: non-OCRC *vs* the control, OCRC *vs* the control and non-OCRC *vs* OCRC, all *P* < 0.001, n = 160, 156, 156 respectively). **(B)** Serum CEA (Kruskal–Wallis test: non-OCRC *vs* the control *P* < 0.001; OCRC *vs* the control *P* < 0.001 and non-OCRC *vs* OCRC *P* > 0.05, n = 158, 99, 99 respectively). **(C)** Serum CA125 (Kruskal–Wallis test: non-OCRC *vs* the control *P* < 0.001; OCRC vs the control *P* < 0.001 and non-OCRC *vs* OCRC *P >*0.05, n = 157, 115, 115 respectively). **(D)** Serum CA19-9 (Kruskal–Wallis test: non-OCRC *vs* the control *P* < 0.01; OCRC *vs* the control *P* < 0.01 and non-OCRC *vs* OCRC *P* > 0.05, n = 152, 105, 99 respectively). **P < 0.01, ***P < 0.001, NS, no statistical significance.

The total number of people in this study is 472, including 156 patients with non-OCRC (57 women and 99 men, age: 63.39 ± 9.53 years), 156 patients with OCRC (50 women and 106 men, age: 62.95 ± 9.31 years) and a control group of 160 healthy people (60 women and 100 men, age: 62.01 ± 5.09 years). The three groups were comparable in terms of age and sex (both *P* > 0.05). The median serum CXCL7 concentration was 0.98 (IQR: 0.75–1.26) ng/ml in control group. The median serum CXCL7 concentration was 1.53 (IQR: 1.14–1.86) ng/ml in non-OCRC, and 1.87 (IQR: 1.50–2.23) ng/ml in the OCRC group. The median concentrations of CXCL7 in non-OCRC and OCRC were both higher than that in controls (both *P* < 0.05). Importantly, the median serum concentration of CXCL7 in OCRC was also higher than the median in non-OCRC (*P* < 0.001; **Figure 1A**). The comparison of the concentration in terms of CEA, CA125, and CA19-9 were all higher than controls in CRC patients (both in non-OCRC and OCRC patients, all *P* < 0.05; **Figures 1B–D**); however, the comparison had no significant difference between non-OCRC and OCRC groups (**Supplementary Table 1**). Patients’ characteristics in non-OCRC and OCRC groups are shown in **Supplementary Table 2**. The difference was only found in T stage between non-OCRC and OCRC groups (*P* = 0.023), no other significant differences were found between two groups.

### Associations Between Serum Levels of CXCL7 and Clinical Pathological Characteristics in OCRC Patients

Associations of serum levels of CXCL7 in OCRC patients referring to age, sex, tumor size, histological type, location, and TNM stage were progressively conducted among all 156 OCRC patients. The median serum CXCL7 concentrations were 1.68 (IQR: 1.34–1.91) ng/ml, 1.91 (IQR: 1.63–2.22) ng/ml, and 1.93 (IQR: 1.54–2.42) ng/ml in N0–N2 subgroups respectively. The results showed that serum CXCL7 expression was elevated in according to the lymph node metastasis stages (N0–N2, r = 0.195, *P* = 0.015). The median serum CXCL7 concentrations were 1.64 (IQR: 1.34–1.90) ng/ml, 1.92 (IQR: 1.56–2.29) ng/ml, and 1.99 (IQR: 1.63–2.38) ng/ml in different TNM stages from I–II to IV. The correlation between serum CXCL7 and TNM stages was statistically significant (r = 0.217, *P* = 0.006). The data showed that no significant correlation between CXCL7 and other pathological characteristics in OCRC patients (all *P* > 0.05, **Table 1**).

**Table 1:** Level of serum CXCL7 related to different clinical pathological characteristics in OCRC patients.

Variable		No.(%)	Median (IQR)	r	P value
Age, year	≤60	54	1.83(1.49–2.23)	0.012	0.881
	>60	102	1.87(1.50–2.26)		
Sex	Male	106	1.82(1.50–2.21)	0.032	0.694
	Female	50	1.90(1.48–2.33)		
Tumor size, cm	≤4	92	1.80(1.49–2.21)	0.065	0.419
	>4	64	1.92(1.53–2.26)		
Histological type	Well	12	1.89(1.69–2.27)	0.101	0.221
	Moderately	112	1.81(1.43–2.20)		
	Poor	32	2.08(1.56–2.33)		
Location	Right	36	1.81(1.63–2.08)	0.005	0.955
	Left	120	1.85(1.45–2.26)		
T stage	2	12	1.86(1.47–1.96)	0.133	0.098
	3	34	1.71(1.34–2.29)		
	4	110	1.89(1.54–2.27)		
N stage	0	45	1.68(1.34–1.91)	0.195	0.015
	1	60	1.91(1.63–2.22)		
	2	51	1.93(1.54–2.42)		
M stage	0	132	1.81(1.49–2.22)	0.120	0.135
	1	24	2.06(1.66–2.39)		
TNM stage	I–II	41	1.64(1.34–1.90)	0.217	0.006
	III	89	1.93(1.59–2.34)		
	IV	26	2.08(1.63–2.40)		

### Diagnostic Efficiency of Serum CXCL7 in OCRC and Non-OCRC Patients

**Figure 2:**
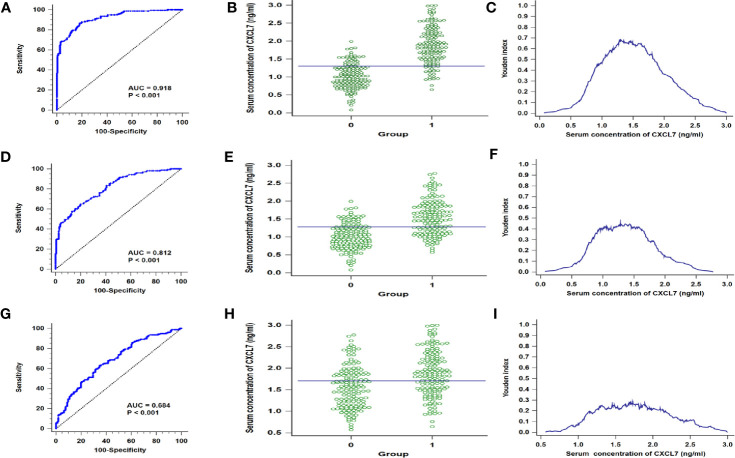
ROC curves for distinguishing the OCRC and non-OCRC patients from controls referring to serum CXCL7. **(A–C)** in discovery cohort. **(A)** AUC in OCRC patients. **(B)** Interactive dot diagram in OCRC patients. **(C)** Youden index in OCRC patients. **(D–F)** AUC, Interactive dot diagram and Youden index in validation cohort of OCRC patients. **(G–I)** ROC curves for OCRC and non-OCRC patients. *Groups: **(B, E)** 0 = Controls, 1 = OCRC group, **(H)** 0 = non-OCRC group, 1 = OCRC group.

According to the comparison of serum CXCL7 in OCRC and controls, the data indicated that serum CXCL7 may be used as a diagnostic biomarker for OCRC patients. Then 316 people were randomly divided into discovery cohort and validation cohort. The discovery cohort had 189 subjects, including 93 OCRC patients and 96 controls, meanwhile the rest 127 people were allocated into validation cohort (63 OCRC patients and 64 controls). Next, the diagnostic test was conducted in the discovery cohort and validation cohort. ROC curve results showed that the AUCs were 0.899 in discovery cohort and 0.942 in validation cohort (**Supplementary Figure 1**), which indicated CXCL7 may be a potential biomarker for OCRC diagnosis. In order to better explore the efficiency of CXCL7 in OCRC diagnosis, all subjects were introduced into analysis, and the results of sensitivity and specificity were evaluated by ROC curve analysis. The AUC of CXCL7 was 0.918 (95% CI: 0.882**–**0.945; *P* < 0.001, **Figure 2A**). The cutoff value was 1.30 ng/ml (**Figures 2B, C**) with a sensitivity of 86.54% and a specificity of 81.87%. Meanwhile the diagnostic test was performed between non-OCRC patients and the controls, the AUC of CXCL7 was 0.812 (95% CI: 0.765**–**0.854; *P* < 0.001, **Figure 2D**), which was lower than OCRC group. The sensitivity, specificity, and cutoff value were shown in **Figures 2E, F** respectively. Furtherly, the diagnostic test was performed between OCRC and CRC patients, the AUC of CXCL7 was 0.684 (95% CI: 0.629**–**0.735; *P* < 0.001; sensitivity: 62.82%, specificity: 65.38%; cutoff value: 1.70 ng/ml; **Figures 2G–I**).

### Diagnostic Efficiency of Serum CXCL7 in Different N Subgroups of OCRC Patients

**Figure 3:**
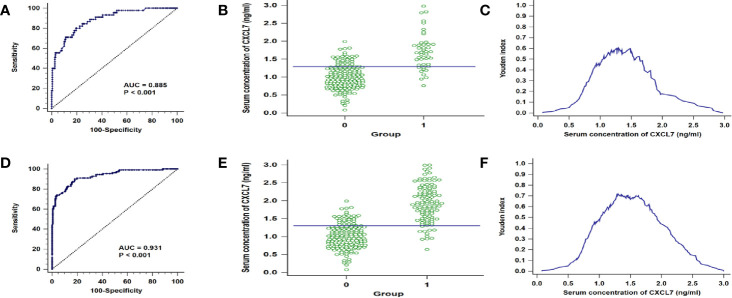
ROC curves in different N subgroups of OCRC patients. **(A)** AUC in N0 patients. **(B)** Youden index in N0 patients. **(C)** Interactive dot diagram in N0 patients. **(D–F)** AUC, Youden index and Interactive dot diagram in N1–2 OCRC patients in order. *Groups: 0 = Controls, 1 = OCRC group.

Based on the results that the levels of CXCL7 were different significances in N0 and N1**–**2 subgroups, the N0 and N1**–**2 OCRC patients were used for analysis respectively. The AUC of CXCL7 was 0.885 (95% CI: 0.833**–**0.925; *P* < 0.001; sensitivity: 80.00%, specificity: 80.62%; cutoff value: 1.29 ng/ml; **Figures 3A–C**) in N0 group; the AUC of CXCL7 was 0.931 (95% CI: 0.894**–**0.958; *P* < 0.001; sensitivity: 90.09%, specificity: 81.87%; cutoff value: 1.30 ng/ml; **Figures 3D–F**) in N1**–**2 group.

### Comparison of Diagnostic Efficiency of CXCL7 and Other Three Markers in OCRC and Non-OCRC Patients

Diagnostic efficiency of CXCL7, CEA, CA125, and CA19-9 was also brought into comparison. Among the five indicators in OCRC patients, the AUC of CXCL7 was maximal; and the AUCs of CEA, CA125 and CA19-9 were, respectively, 0.786 (95% CI: 0.731–0.835, *P* < 0.001, **Supplementary Figure 2A**), 0.657 (95% CI: 0.579–0.713, *P* < 0.001, **Supplementary Figure 2B**) and 0.614 (95% CI: 0.551–0.674, *P* = 0.002, **Supplementary Figure 2C**). In non-OCRC patients, the AUCs of CEA, CA125, CA19-9, and CA724 were, respectively, 0.784 (95% CI: 0.729–0.833, *P* < 0.001, **Supplementary Figure 2D**), 0.666 (95% CI: 0.607–0.722, *P* < 0.001, **Supplementary Figure 2E**), and 0.611 (95% CI: 0.548–0.671, *P* = 0.003, **Supplementary Figure 2F**). Deeply, the diagnostic test was performed between OCRC and CRC patients by using these biomarkers to make a thorough exploration on the ability of distinguishing OCRC and CRC patients. Out of our expectation, the results showed that CXCL7 had a higher AUC than other markers (**Supplementary Figures 2G–I**).

### The Levels of Serum CXCL7 in Postoperative OCRC Patients

**Figure 4:**
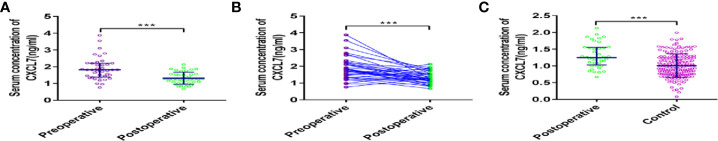
Analysis of serum CXCL7 in postoperative OCRC patients. **(A)** Comparison of the expression levels of serum CXCL7 (Postoperative *vs* Preoperative). **(B)** Comparison of the expression levels of serum CXCL7 (Postoperative *vs* Preoperative, matched by 1:1). **(C)** Comparison of the expression levels of serum CXCL7 (Postoperative *vs* Control, matched by 1:1). ***P < 0.001.

In order to know the concentration change of CXCL7 in preoperative and postoperative OCRC patients, a total of 40 OCRC patients in diagnostic test were selected as participants to detect the levels of CXCL7 after one month of resection operation. Seven women and 33 men were involved, including Stage I one patient, Stage II 16 patients, Stage III 17 patients, Stage IV six patients. The concentration of CXCL7 in postoperative OCRC patients was 1.25 (IQR: 1.03–1.56) ng/ml, which was descended compared with the level of preoperative OCRC patients (1.81, IQR: 1.38–2.20 ng/ml, *P* < 0.05, **Figures 4A, B**). Although the concentration of CXCL7 has decreased after resection operation, the levels of CXCL7 were still higher than controls (*P* < 0.05, **Figure 4C**). Therefore, the concentration of CXCL7 in postoperative OCRC was higher than normal people and lower than preoperative patients.

### Upregulation of Serum CXCL7 Predicted the Poor Prognosis in OCRC Patients

**Figure 5:**
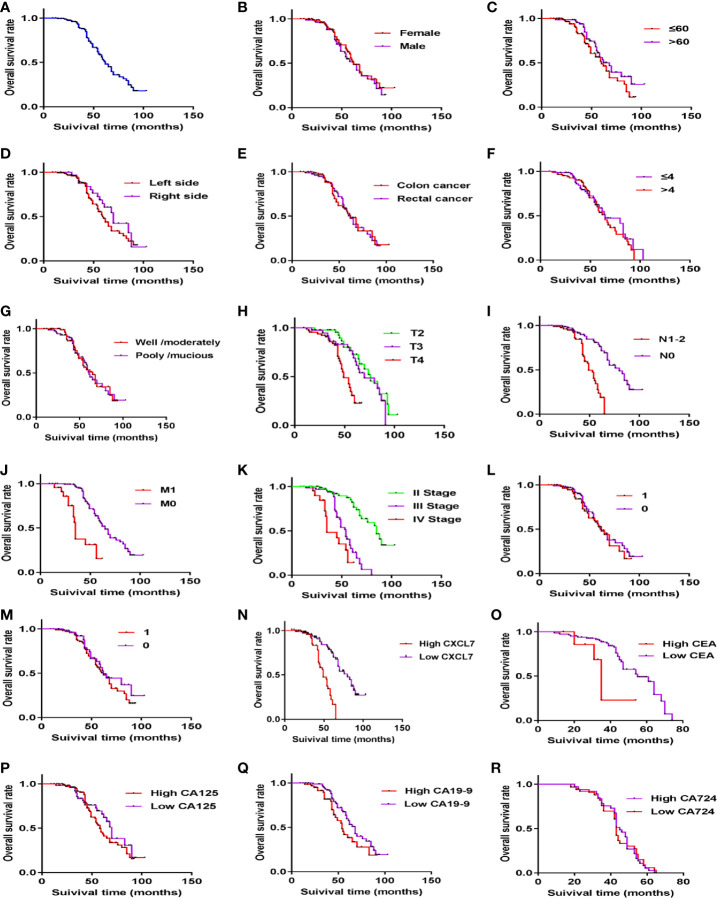
Kaplan–Meier curves of OCRC patients showing the overall survival time after primary tumor resection. **(A)** Survival time of all OCRC patients. **(B)** Survival time in different gender subgroups (*P* > 0.05). **(C)** Survival time in different age subgroups (*P* > 0.05). **(D)** Survival time in different locations (*P* > 0.05). **(E)** Survival time in Colon and Rectum of OCRC patients (*P* > 0.05). **(F)** Survival time in Tumor size of OCRC patients (*P* > 0.05). **(G)** Survival time in different Histological type (*P >* 0.05). **(H-K)** Survival time in different T stages, N stage, M stage, and TNM stage one by one (all *P* < 0.05). **(L, M)** Survival time in of OCRC patients related to Vascular invasion **(L)** and Nerve invasion(M) (1: yes, 0: no; all *P* > 0.05). **(N)** Survival time in different CXCL7 groups (high *vs* low in serum; *P* < 0.05). **(O)** Survival time in different CEA groups (high *vs* low in serum; *P* < 0.05). **(P–R)** Survival time in different CA125, CA19-9 and CA724 subgroups respectively (high *vs* low in serum; all *P* > 0.05).

Above all the results of the diagnosis tests, serum CXCL7 may be a diagnostic biomarker of OCRC. Further, to evaluate the prognostic value of CXCL7 in OCRC patients, 155 patients were followed up after surgery (one people lost follow-up) for getting the survival state and time. The median survival time of all 155 OCRC patients was 62.00 (95% CI: 55.28**–**68.72) months and the 5-year overall survival rate of the patients was 51.80% (**Figure 5A**). There were significantly differences in OS in terms of T stage (HR: 1.688, 95% CI: 1.222**–**2.332, *P* = 0.001; **Figure 5H**), N stage (HR: 2.825, 95% CI: 1.555**–**5.132, *P* =0.002; **Figure 5I**), M stage (HR:4.588, 95% CI: 2.429**–**8.555, *P* < 0.001; **Figure 5J**), TNM stage (HR:3.262, 95% CI: 2.278**–**4.671, *P* < 0.001; **Figure 5K**), CXCL7 (HR:4.804, 95% CI: 2.672**–**8.676, *P* < 0.001; **Figure 5N**), and CEA (HR:2.696, 95% CI: 1.625**–**4.473, *P* < 0.001; **Figure 5O**). The details were shown in **Table 2**. However, no statistical differences were found in other factors (**Figures 5B-5G, 5L, 5M, 5P-5R**, and **Table 2**; all *P* > 0.05). Multivariate analysis showed that high CXCL7 in serum was independent factor associated with poor OS of OCRC patients (HR = 2.216, 95% CI: 1.069**–**4.593, **Table 2**, *P* = 0.032).

**Table 2:** Cox proportional hazard regression analysis on the expression of serum CXCL7 in OCRC patients.

Variable		3-year OS(%)	5-year OS(%)	Univariate analysis		Multivariate analysis	
				HR(95%CI)	*P* value	HR(95%CI)	*P* value
Gender	Male	72.4	48.2	1.164(0.729–1.857)	0.525		
	Female	76.4	56.5				
Age (years)	≦60	74.2	56.1	0.694(0.432–1.116)	0.132		
	>60	72.3	39.2				
Location	Right	87.5	64.7	0.713(0.407–0.251)	0.225		
	Left	87	70.6				
Colorectal	Colon	88.3	50.6	0.967(0.606–1.543)	0.889		
	Rectum	89.1	49.9				
Tumor size (cm)	≤4	86.9	51.0	1.032(0.644–1.654)	0.895		
	>4	88.6	52.3				
Histological type	Well/moderately	86.3	52.9	1.012(0.634–1.614)	0.960		
	Poor/mucinous	89.7	50.7				
T stage	T2	88.1	71.0	1.688(1.222–2.332)	0.001	1.338(0.947–1.888)	0.098
	T3	90.1	56.3				
	T4	81.9	17.6				
N stage	N0	89.5	58.2	2.825(1.555–5.132)	0.002	1.398(0.730–2.678)	0.312
	N1–2	81.4	10.1				
M stage	M0	95.0	56.2	4.588(2.429–8.555)	<0.001	1.960(0.728–5.277)	0.183
	M1	37.5	15.6				
TNM stage	II	92.5	81.9	3.262(2.278–4.671)	<0.001	2.226(1.168–4.244)	0.015
	III	91.7	29.1				
	IV	48.3	14.1				
Vascular invasion	1	84.0	52.5	0.872(0.532–1.416)	0.581		
	0	91.2	51.7				
Nerve invasion	1	88.0	48.4	1.226(0.745–2.017)	0.418		
	0	92.0	47.5				
CXCL7	High	83.1	15.9	4.804(2.672–8.676)	<0.001	2.216(1.069–4.593)	0.032
	Low	93.6	74.2				
CEA	High	82.8	28.6	2.696(1.625–4.473)	<0.001	1.664(0.975–2.840)	0.062
	Low	93.5	67.5				
CA125	High	83.3	65.7	0.766(0.459–1.278)	0.299		
	Low	91.4	45.1				
CA19-9	High	91.3	38.1	1.619(1.000–2.622)	0.055		
	Low	93.1	59.2				
CA724	High	86.6	47.0	1.186(0.736–1.911)	0.485		
	Low	91.0	54.7				

## Discussion

Intestinal obstruction is a common clinical complication of colorectal cancer. Once intestinal obstruction occurred, the treatment methods may be very troublesome, meanwhile the prognosis would be unsatisfactory even a large number of treatment measures had been implemented for OCRC patients (8, 25). Many studies have proved that OCRC patients have worse prognosis than non-OCRC patients and often suffered from an advanced stage (8, 9, 26–28). Therefore, it is so vital for OCRC patients with an early diagnosis as soon as possible and the overall survival time would be elevated with timely treatment. In this study, the serum CXCL7 was measured and analyzed by plotting ROC curves to make a thorough exploration of the ability in OCRC diagnosis. Then, the CXCL7 was used to ascertain the relation between the long-time outcomes and prognostic factors.

CXCL7 belongs to ELR^+^ CXC chemokines and functions binding to its receptor CXCR2 (29), and has a prominent effect on immune response by recruiting neutrophils to the sites of vascular injury (30, 31). CXCL7 has been proved to be a vital tumor microenvironment regulator in several cancers and a potential biomarker in diagnosis of cancers (16, 24, 28). The level of CXCL7 was detected in early lung cancer and was a potential marker with an AUC of 0.64 (32). Other study also focused on the efficiency of CXCL7 in lung diagnosis with an AUC of 0.83, which suggested that CXCL7 may be a diagnostic biomarker in lung cancer (33). In our study, the concentration of serum CXCL7 is higher in OCRC patients than controls. Further, CXCL7 was also higher in OCRC patients than non-OCRC group. The comparison among three groups supported our assumption that CXCL7 in serum could make a distinction between OCRC, non-OCRC and controls to some extent. For clearly displaying the results for diagnostic tests, we also made ROC curves graphs, Youden index graphs and Interactive dot diagram together. As the results shown, the CXCL7 in serum of OCRC patients had an AUC of 0.918 when compared with controls, and AUC of 0.684 when compared with non-OCRC, which were higher than other three markers. It illustrated CXCL7 could be utilized as a biomarker for detecting OCRC.

In this study, the patients with high levels of serum CXCL7 had significantly poorer oncologic outcomes in OCRC patients. Overexpression of CXCL7 is associated with poor prognosis in several cancers and connected to tumor growth, invasion, migration and angiogenesis by motivating the PI3K/AKT/mTOR signaling pathways (34, 35). The signaling pathway of AKT existed in all cells of people and participated in many metabolic processes, such as cell growth, apoptosis, migration, *etc.* (36, 37 *).* CXCL7 can also play its role through Ras/Raf/mitogen-activated protein kinase (MAPK) signaling pathways associated with tumor angiogenesis (38). CXCL7 could accelerate tumor metastasis *via* regulating the expression of VEGF-C/D in breast cancer (39). The ERK pathway also can be accelerated by CXCL7-CXCR2/CXCR1 axis (40). Thus, CXCL7 may be a factor in the occurrence process of OCRC by activating AKT and other signaling pathway, leading to a poor survival outcome in OCRC patients. The serum CXCL7 was just correlated with TNM stages in all pathological characteristics in OCRC patients, so we assumed that CXCL7 could be a selected marker for predicting long-time outcomes of OCRC patients. By using univariate and multivariate Cox proportional hazard regression (41), it proved that CXCL7 in serum was an independent prognostic factor with worse outcomes compared with low concentration of CXCL7 in OCRC patients, and this result was consistent with previous studies that the overexpression of CXCL7 was a predicted signal of poor prognosis in CRC patients (42).

In order to comprehensively explore the efficiency of CXCL7 in diagnosis and prognosis of OCRC patients, some details in this study will continue to be explored for deeply interpreting the ability of CXCL7. First, the number of OCRC patients should be expanded with patients of different regions and various races. Then, habits and custom information of patients, such as smoking, drinking, exercise, *etc*., should be collected once the patients were enrolled in this study. Next, the levels of CXCL7 should continuously detected if possible, and the preoperative levels of CXCL7 in OCRC patients should be compared with the concentration of CXCL7 in postoperative patients, thus the role of CXCL7 may be illustrated in the process of OCRC and provide more information about the connection with other factors. More importantly, the molecular mechanism of CXCL7 may be explored, and the biological functions of CXCL7 will be studied in OCRC patients, someday in the future. Overall, this is the first time to quantitatively measure serum CXCL7 for exploring the efficiency in diagnosis and prognosis of OCRC patients. The increased levels of CXCL7 in serum were associated with the TNM stages and poor prognosis, and the results showed that serum CXCL7 may be a potential biomarker both in diagnosis and prognosis for OCRC patients. This study also expands our understanding of the roles of small molecule cytokines in cancer diagnosis and prognosis.

## Data Availability Statement

The raw data supporting the conclusions of this article will be made available by the authors, without undue reservation.

## Ethics Statement

The studies involving human participants were reviewed and approved by Institutional Research Ethics Committee of the Affiliated Hospital Jiangnan University. The patients/participants provided their written informed consent to participate in this study.

## Author Contributions

LL, LZ, and LX conceived the study. LL, TZ were in charge of performing the experiments and analyzing the data. LL and TZ were responsible for supervision, writing and editing the manuscript. All authors contributed to the article and approved the submitted version.

## Funding

This study was supported by the National Natural Science Foundation of China (No. 81372375). 

## Conflict of Interest

The authors declare that the research was conducted in the absence of any commercial or financial relationships that could be construed as a potential conflict of interest.

## Abbreviations

CRC, colorectal cancer; OCRC, obstructive colorectal cancer; CEA, carcinoembryonic antigen; CA125, carbohydrate antigen 125; CA19-9, carbohydrate antigen19-9; ELISA, enzyme-linked immunosorbent assay; M, Median; IQR, interquartile range; ROC, receiver operating characteristic; AUC, area under the curve; 95% CI, 95% confidence interval; Youden index, sensitivity + specificity-1; TNM, tumor-node-metastasis; T, depth of tumor invasion; N, nodal involvement; M, distant metastasis; OS, overall survival time; HR, high risk factor.
