# Supplementary figures and images for: Serum Chemokine CXCL7 as a Potential Novel Biomarker for Obstructive Colorectal Cancer
Source: Front Oncol. 2021 Feb 10;10:599363. doi: 10.3389/fonc.2020.599363 (PMC7902867; doi:10.3389/fonc.2020.599363)

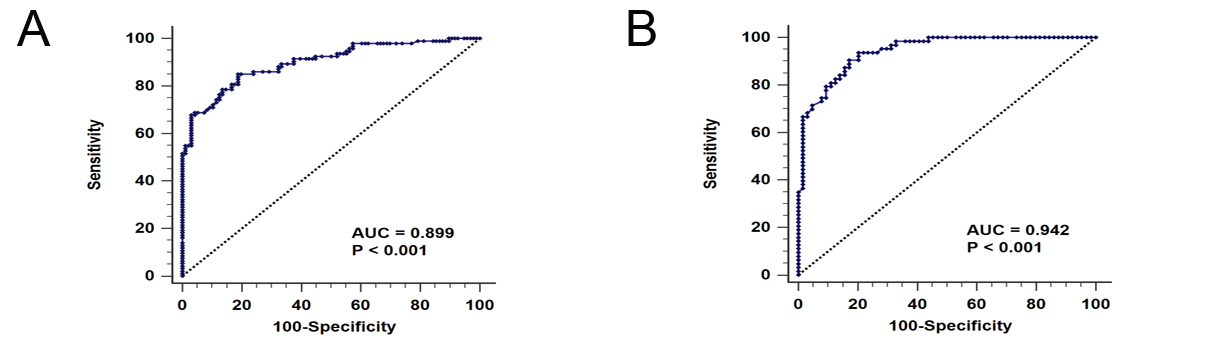

Supplement: Supplementary file 1 [file Image_1.tif]

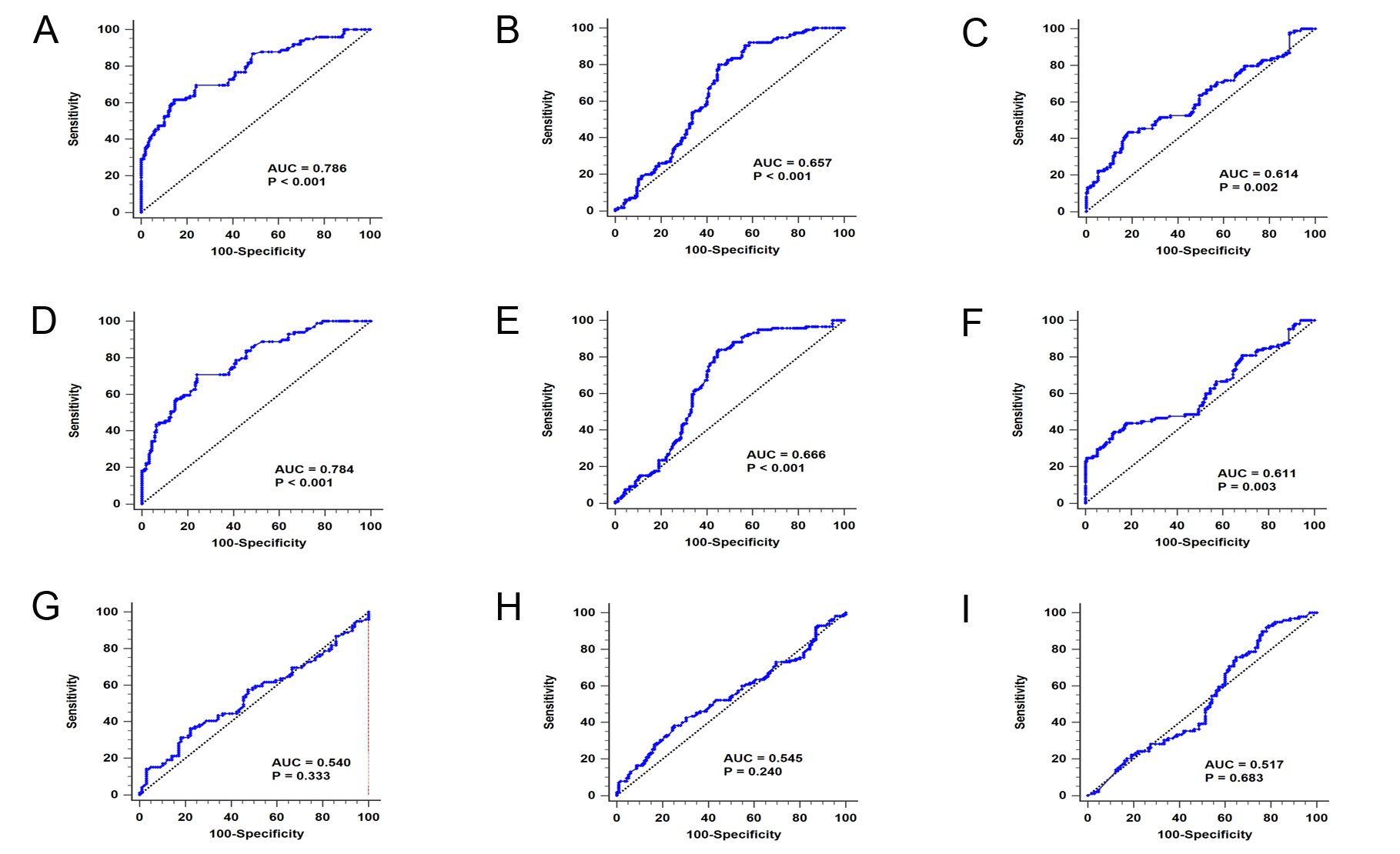

Supplement: Supplementary file 2 [file Image_2.tif]
